# Exploration on the optimization of occupational injury and employment protection of takeout workers in the context of public health

**DOI:** 10.3389/fpubh.2023.1115128

**Published:** 2023-02-28

**Authors:** Qifan Wang, Qingyu Liu, Tianyi Zhu

**Affiliations:** College of Political Science and Law, Jiangxi Normal University, Nanchang, Jiangxi, China

**Keywords:** occupational injury, employment protection optimization, takeaway employees, public hygiene, analytic hierarchy process

## Abstract

With the acceleration of the pace of urban life and the development of information technology, the takeout industry has emerged as the times require, which obtains intermediate costs by distributing goods to consumers. People pay more and more attention to public health, which requires takeout workers to drive as fast as possible to ensure the quality and safety of goods, but it also makes takeout workers suffer from various occupational injuries, such as car accidents, stomach diseases caused by eating disorders and long-term psychological pressure. This paper optimized the employment protection of takeout workers in combination with their professional characteristics. This paper used the analytic hierarchy process (AHP) to analyze the indicators that can evaluate the optimization effect of employment protection for takeout workers, and compared the occupation of takeout workers before and after employment protection. The experimental results showed that in Meituan takeout, the rationality of the average delivery management system before and after the optimization of employment protection was 47.2 and 64.4%, respectively; in ELEME takeout, the rationality of the average takeout distribution management system before and after the optimization of employment protection was 55.0% and 69.8%, respectively. Therefore, in the context of public health, the implementation of social security, employment relationship and optimization of service evaluation mechanism for outbound sales personnel can effectively improve the rationality of the delivery management system.

## 1. Introduction

People's living standards are improving, and their lifestyles have undergone tremendous changes. Many young people, Baoma and migrant workers are more inclined to obtain the goods they need through special delivery due to high work pressure or lack of conditions to cook. With the continuous development of Internet technology, and the growing demand for people to sell and distribute, the takeout industry came into being. Taking out belongs to the service industry. It mainly refers to the services that businesses provide all the goods that can be delivered, including food distribution, water delivery and door-to-door repair. The takeout workers provided convenience for people's lives, which accelerated the development of the tertiary industry and met people's needs in life. However, the takeout employees need to deliver goods as quickly as possible, which leads to frequent accidents of takeout employees, and there is no relevant occupational risk guarantee for the injury of takeout employees. When the takeout employees fail to complete the specified quantity, they would be dismissed. The takeout employees lack employment protection. In recent years, the takeout industry has developed rapidly. According to relevant statistics, China has the largest number of takeout workers in the world. In 2020, there were 16 million takeout workers, 90% of whom were men and 10% are women. The takeout workers have promoted the economic development, but their own occupational injuries are from many sources, and their occupations cannot be guaranteed stably. By optimizing the employment protection of takeout employees, reducing the occupational injury of takeout employees, and strengthening the employment relationship between takeout employees and the takeout platform, the employment of takeout employees can be guaranteed. The takeout industry is a new type of business, which protects the labor rights and interests of takeout personnel through targeted occupational protection. Therefore, this paper has research significance.

The rapid development of Internet technology has created many emerging industries, and many people have conducted in-depth research on the occupational injuries of people in emerging industries. Fischer (1) analyzed the work and rest situation and occupational risks of the takeout workers, and the research showed that: the long-term physical, mental and muscle fatigue of the takeout workers is the main reason for the occupational injuries of the takeout workers. Kearney et al. used meta-analysis to systematically analyze the risks of physical and psychological injuries affecting nursing staff. Through the investigation of occupational injuries among 1,000 nursing staff, it was found that the risk of occupational injuries among nursing staff was increasing year by year (2). New-Aaron (3) made detailed statistics on the occupational injuries and deaths of workers in the United States. The results showed that 44% of agricultural injuries were fatal. Clouser interviewed Latino farm workers to analyze the reasons that affect the occupational injury risk of Latino farm workers. Among them, work pressure, unfair supervisors and inability to communicate fluently in Spanish language were the main reasons for Latino farm workers' occupational injury (4). By analyzing the occupational injuries of employees, the main causes of occupational injuries of employees can be calculated. However, there is a lack of targeted optimization of employment protection for occupational injuries of employees.

With the improvement of people's living standards, more and more attention has been paid to the employment protection of the bottom personnel. Many people have conducted in-depth research on the optimization of the employment protection of takeout workers. Dessaint et al. (5) pointed out that the implementation of the employment protection policy effectively prevented the enterprises from arbitrarily cutting jobs and provided employment security for employees. Passaretta et al. (6) analyzed the employment of employees in the European labor market. Long term labor contracts can ensure stable employment of workers. Vornholt analyzed the occupational injuries and employment status of the disabled. The study pointed out that the disabled are important labor resources in the labor force, and they need to be optimized for employment protection to stabilize their employment (7). Although the optimization of employment protection for workers can improve the occupational safety and stability of workers, it is rare to compare the employment situation of workers before and after the optimization of employment protection.

The appearance of takeout platform has satisfied the development of urban fast-paced life and provided employment for a large number of urban migrant workers, but the occupational injury of takeout employees is huge. This paper optimized the employment protection according to the characteristics of occupational injuries of takeout workers, and compared the employment protection of takeout workers before and after the optimization. The results showed that the optimization of employment protection can reduce the psychological pressure of takeout workers.

## 2. Optimization method of employment protection for takeout employees

**Figure 1:**
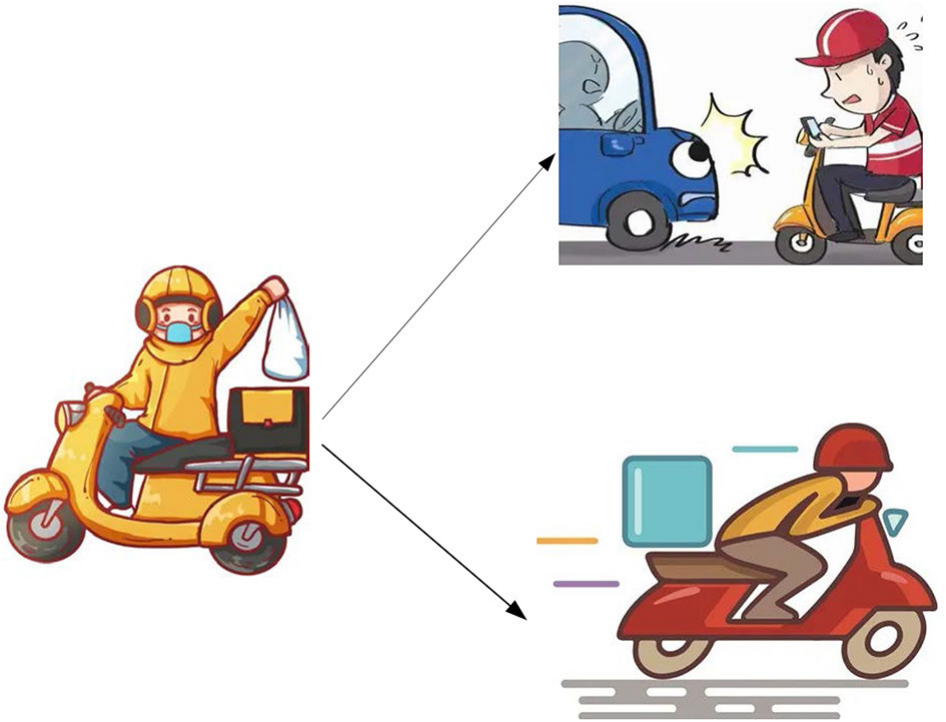
Examples of occupational injuries of takeout employees.

In order to cope with the fast-paced life demand, many new business forms have emerged under the development of the Internet. The new business forms are characterized by flexible employment and simple work content, which provide employment options for a large number of people who do not know what to do. It has attracted a large number of practitioners. The employees of the new business include delivery personnel, express delivery personnel and online car hailing drivers, which provide life services for the development of society. Among them, takeout workers account for the largest proportion. The way of work liberalization enables takeout workers to arrange their working hours flexibly, but the vast majority of takeout workers compete with time, which also indirectly leads to occupational injuries of takeout workers. The examples of occupational injuries of takeout workers is shown in Figure 1.

Figure 1 shows examples of occupational injuries of takeout workers. All takeout workers compete with time in the process of delivering meals. Falling down or sudden death due to high fatigue are common occupational injuries of takeout workers.

With the continuous development of information technology, the takeout platform led by Meituan and ELEME quickly occupied the catering market, which provide a convenient life for urban residents. In addition, it provides businesses with more opportunities, and also provides more urban migrant workers with a job opportunity. However, with the development of takeout platforms, the competition between takeout platforms is becoming more and more fierce, and the takeout management system is becoming more and more rigorous. As a service worker, the takeout workers should meet all the requirements of customers and ensure the delivery quality and speed of goods within the specified time. The strict management of takeout employees on the takeout platform makes it very easy for takeout employees to cause occupational injuries in the struggling working environment, which is an extremely unfair system for takeout employees (8).

### 2.1. Public health

Public health is to analyze the population, and study the law of the impact of environment on health through mature medical means, so as to analyze the relationship between social environment and human health, and find out ways to improve population health (9, 10). Public health measures can achieve the goal of controlling diseases and protecting people's health.

Environmental sanitation is to analyze the impact of community environment on human health, and determine the main environmental factors that affect people's lives by subdividing their living environment. On the basis of making full use of beneficial environmental factors and controlling harmful environmental factors, it puts forward hygiene requirements and preventive measures, which can improve human health and the health level of the whole population.

Occupational health mainly analyzes the impact of occupational environment on labor health. Through the analysis of different occupational environments, it can create a more comfortable working environment for workers, and try to meet the physical and psychological needs of workers, so that workers can achieve the best state at work.

**Figure 2:**
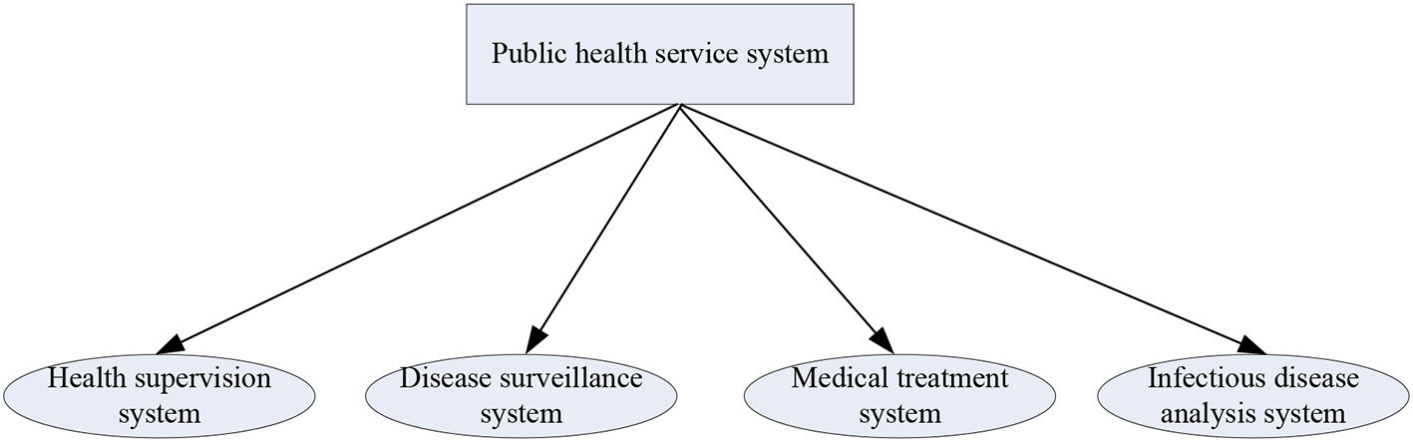
Public health service architecture.

Public health, environmental health and occupational health play a more and more important role in protecting and promoting people's health and preventing diseases, and are the basis of disease prevention and control activities. The public health service architecture is shown in Figure 2.

Figure 2 depicts the public health service architecture. The public health system is divided into four subsystems: health supervision system, disease detection system, medical treatment system, and infectious disease analysis system.

Public health needs to control population health or disease prevalence. It analyzes the distribution of diseases in different populations, time and space, and analyzes and intervenes on the future development trend of diseases (11, 12). It analyzes the impact of living environment and genetic genes on human health, and analyzes the protein genes that affect human health functions from the perspective of biological genes. It analyzes the living environment, daily diet and living rules of the human body, and explores the impact of life factors of the population on human health, so as to propose corresponding protection measures.

In the fast-paced urban life, people often have no time to cook because of the compact work content. For office workers, takeout has become the first choice to solve the problem of eating. Takeout food has become a necessity in people's daily life. Therefore, the hygiene problem of takeout food needs to be attached great importance. Public health has strict rules and regulations for ensuring the hygiene of takeout food.

The takeout workers need to confirm the food temperature when delivering food, and deliver the takeout to consumers when the temperature is kept. In the process of food production, storage and transportation, the staff must pass physical examination to obtain health certificates. The delivery staff participate in the food transportation, so health certificates are also necessary. Take away stores need to be open to the outside world so that consumers have the right to know. At the same time, it is necessary to supervise the takeout stores to ensure food safety.

### 2.2. Optimization of occupational injury and employment protection

Occupational injury refers to the injury or accident suffered by employees in the process of carrying out work or activities related to their occupation (13, 14). As an emerging industry, the takeout industry provides convenience for people's lives and also provides a job for urban migrant workers. However, the problem of occupational injuries among employees is becoming more and more serious (15).

The takeout food industry has developed in recent years. The main job of the takeout workers is to deliver the goods from the merchants to the consumers safely and on time. Such a simple delivery task should not cause frequent occupational injuries. The main reasons for frequent occupational injuries among takeout employees are.

The delivery management system is not comprehensive enough. In some non-meal time, the delivery staff need not rush to deliver, and the traffic environment is also very smooth. However, in the peak meal time, a delivery clerk would send seven to 10 meals in a short time, which forces the delivery clerk to ride fast on the road (16). In addition, takeout riders often violate traffic rules in order to rush, which indirectly leads to accidents among takeout employees.

The working environment is bad, and the threshold of the delivery industry is very low. However, if the delivery clerk wants to earn a considerable economic income, he must work for a long time. The delivery environment is very bad. Generally speaking, the vast majority of consumers order takeout because of the bad weather, so the takeout workers must deliver in the hot, windy and rainstorm environment. Working in such bad conditions also increases the risk of the takeout workers.

The work intensity is high. The takeout workers do not only work at breakfast, lunch and dinner. Many takeout workers work for more than 10 h a day. For such intensive work, takeout workers often cannot eat on time. Many takeout workers have stomach problems of varying degrees.

The psychological pressure is high. As a kind of service industry, the evaluation of consumers would directly affect the work performance of takeout practitioners. Many takeout workers try to seize the time to provide the best service for consumers. However, in the long run, takeout workers are under great psychological pressure. When consumers comment on business trips, takeout workers would not only face fines, but may even face resignation.

**Figure 3:**
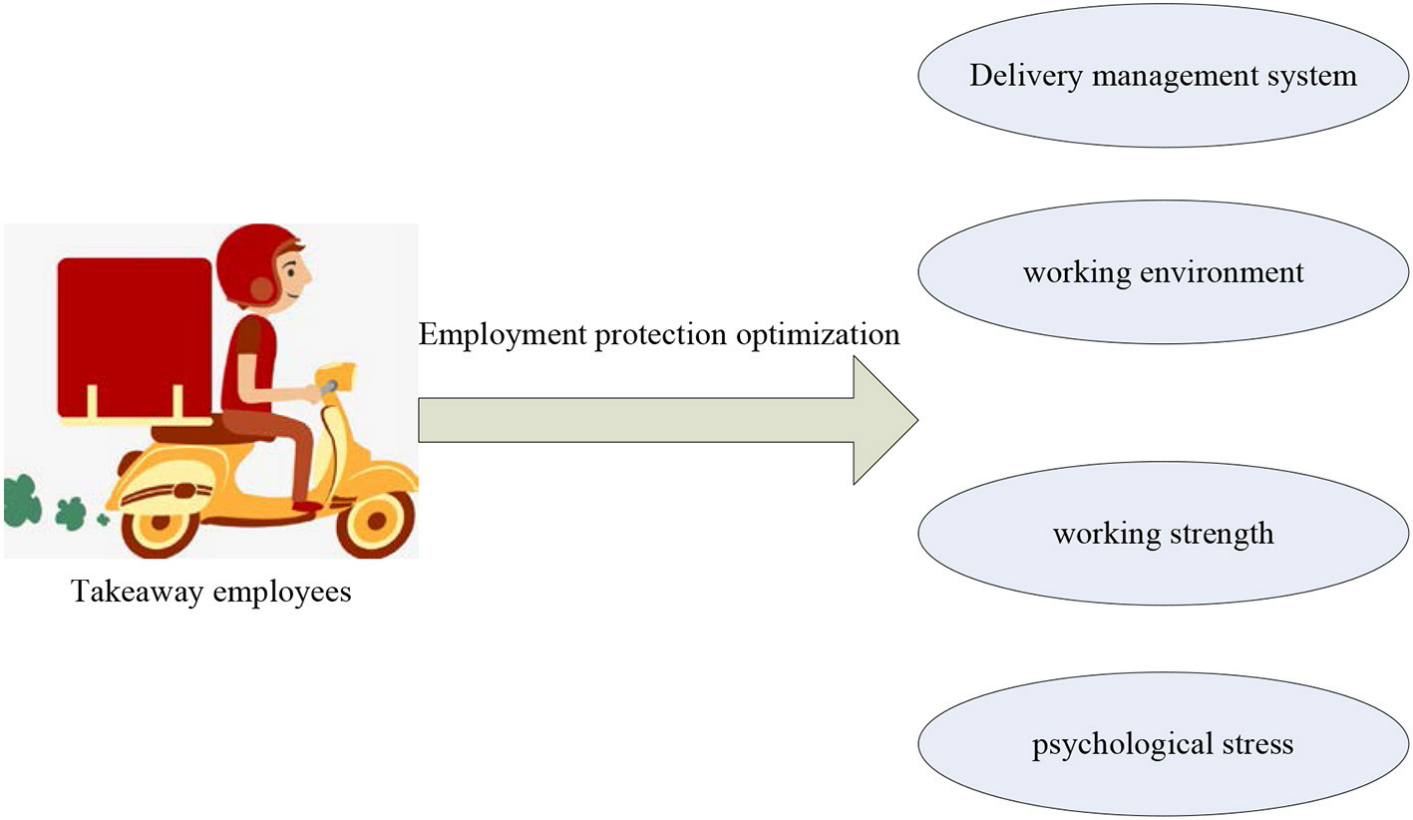
Model diagram of occupational injury and employment protection optimization of takeout employees.

Employment is the result of mutual search between labor supply and demand. The takeout industry has provided a large number of jobs to the society and solved the employment problem of many people. However, the employment relationship between takeout employees and the takeout platform is not stable, and the occupational injury of takeout employees is also closely related to employment protection. It is necessary to optimize the employment protection of takeout workers and improve the employment stability of takeout workers, so as to reduce the frequency of occupational injuries. The optimization model of occupational injury and employment protection of takeout employees is shown in Figure 3.

Figure 3 describes the optimization model of occupational injury and employment protection of takeout workers. Through the optimization of employment protection, the management system of delivery can be adjusted and the working environment can be improved. It can also adjust the work intensity and reduce the psychological pressure of takeout employees.

For the occupational injuries of takeout employees, the following aspects can be optimized for employment protection:

Social security optimization: because of the great mobility of takeout employees, many takeout employees have not received the social insurance paid by the takeout platform, which is very unfavorable for employees engaged in fatigue industries. Therefore, the internship period for takeaway workers can be reduced, and social insurance can be provided for takeaway workers as early as possible.

Employment relationship optimization: generally, the takeout platform employs riders from third-party companies, which makes the employment relationship between the takeout platform and riders unstable. This causes many takeout practitioners to be dismissed by the platform. It is necessary to strengthen the employment relationship between the takeout platform and riders.

Service evaluation mechanism optimization: traditional takeout platforms attach great importance to the evaluation of consumers. Although this is a means to respect the rights and interests of consumers, it is too harsh for takeout practitioners, and some consumers would maliciously give travel evaluations, which seriously affects the performance of takeout workers. The service evaluation mechanism needs to be optimized. The platform should make a correct investigation on the evaluation of bad comments, and should not blame all the takeout practitioners.

### 2.3. AHP

**Figure 4:**
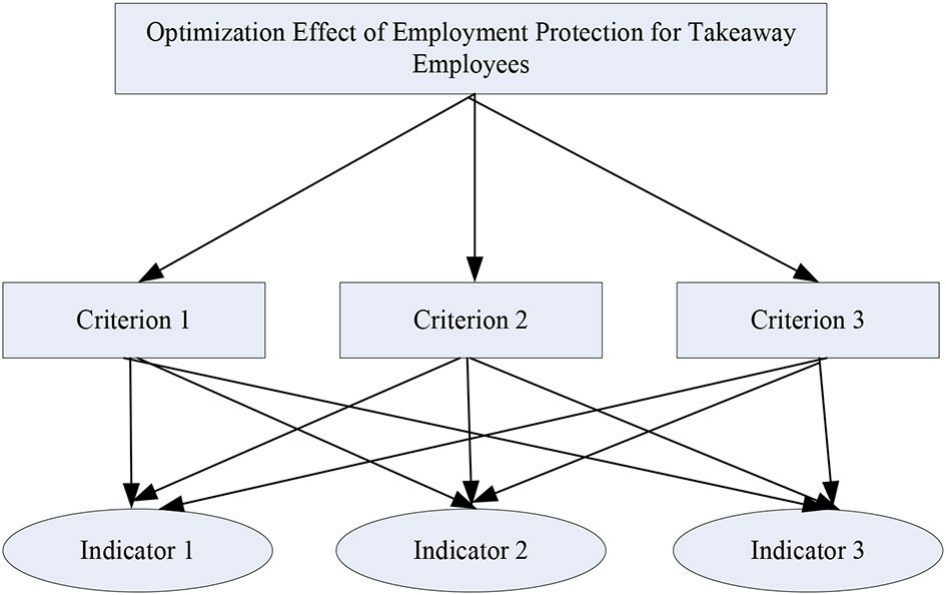
Structure model diagram of AHP.

AHP is an analytical method that combines quantitative and qualitative analysis. It determines the impact indicators by refining the solution to the problem, and then analyzes the indicators (17, 18). AHP can solve complex logic problems, and is very suitable for analyzing the factors that affect the optimization effect of employment protection for takeout workers. The structure model of AHP is shown in Figure 4.

Figure 4 is the structure model of the AHP. The overall structure is divided into three layers. Among them, the criterion layer is a rough analysis of the optimization effect of the employment protection of the selling employees, and the indicator layer is a detailed analysis of the optimization effect of the employment protection of the selling employees.

It is supposed that in the system that affects the optimization effect of employment protection for takeout workers, the indicator layer is *D* = {*d*_1_, *d*_2_, …, *d*_*n*_}, then the equation for the comparison of the impact of any two indicators on the optimization effect of employment protection is:

(1) $$\begin{array}{l} d_{ij}=\frac{d_{i}}{d_{j}}. \end{array}$$

In Equation (1), *d*_*i*_ represents the *i*-th indicator, and *d*_*j*_ represents the *j*-th indicator. The value range of *i* and *j* is {1, 2, ⋯ , *n*}.

All indicators are compared in pairs and a judgment matrix is constructed.

(2) $$\begin{array}{l} Q=\left[\begin{array}{c} \;1\;d_{12}\;\cdots \;d_{1n}\\ d_{21}\;1\;\cdots \;d_{2n}\\ \;\vdots \;\vdots \;\ddots \;\vdots \\ d_{n1}\;d_{\text{n2}}\;\cdots \;1 \end{array}\right]. \end{array}$$

In Equation (2), each column of the matrix can be regarded as the relative weight of the indicator.

Before using the judgment matrix to calculate the relative weight of each indicator in the AHP, it is necessary to analyze the consistency of the judgment matrix (19, 20).

The consistency indicator is calculated as:

(3) $$\begin{array}{l} CI=\frac{t_{\max}-n}{n-1}. \end{array}$$

In Equation (3), CI represents the consistency index, and *t*_max_ represents the maximum eigenvalue of the judgment matrix.

The consistency ratio is expressed as:

(4) $$\begin{array}{l} CR=\frac{CI}{RI}. \end{array}$$

In Equation (4), CR represents the consistency ratio. RI represents the random consistency index corresponding to the judgment matrix.

When *CR* < 0.1, it means that the judgment matrix is consistent, and the weight of the indicator can be calculated by using the judgment matrix (21). When CR ≥ 0.1, it means that the judgment matrix does not have consistency and needs to be re planned.

The relative weight of indicators is expressed as:

(5) $$\begin{array}{l} w_{i}=\frac{d_{1i}+d_{2i}+\cdots +d_{ni}}{n}. \end{array}$$

In Equation (5), *w*_*i*_ represents the relative weight of the *i*-th indicator.

## 3. Experiment on optimization of employment protection for takeout employees

### 3.1. Construction of the optimized evaluation system for the employment protection of takeout employees

The occupational injury of takeout employees is serious, and it is necessary to optimize the employment protection of takeout employees. In order to effectively analyze the effect of the optimization of employment protection, this paper used the AHP to build an evaluation system for the optimization of employment protection, and conducted a questionnaire survey on 200 takeout workers, which mainly investigated the indicators that they thought can evaluate the optimization effect of employment protection. The results of the questionnaire survey are shown in Table 1. The results of the questionnaire will be used as the basis for the analysis of the optimization of the employment protection of takeaway workers.

**Table 1:** Questionnaire results of the evaluation indicators of employment protection optimization.

**Serial number**	**Evaluating indicator**	**Number of people (persons)**	**Percentage**
1	Rationality of delivery management system	56	28%
2	Comfort of working environment	48	24%
3	Working strength	52	26%
4	Psychological stress	44	22%

Table 1 describes the questionnaire results of the evaluation indicators of employment protection optimization. A total of four indicators have been calculated, of which the rationality of the delivery management system accounted for 28% at the highest; the proportion of work intensity was 26%, and the proportion of psychological stress was at least 22%.

Since the indicators analyzed in the questionnaire cannot determine the weight of each indicator, the weight of each indicator is determined by constructing a judgment matrix. The weight of the optimization evaluation index of employment protection is shown in Table 2.

**Table 2:** Weights of employment protection optimization evaluation indicators.

**Target**	**Evaluating indicator**	**Index weight**
Optimization effect of employment protection	Rationality of delivery management system	32%
	Comfort of working environment	28%
	Working strength	22%
	Psychological stress	18%

Table 2 describes the relative weights of each indicator. The rationality of the delivery management system accounted for the highest weight of 32%; the weight of work environment comfort was 28%, and the weight of psychological stress was at least 18%. Since the weight difference of these four indicators is not very large, these four indicators can all be used as indicators to evaluate the optimization effect of employment protection for takeout workers.

### 3.2. Experimental design for optimization of employment protection of takeout employees

In order to effectively analyze the effect of the optimization of employment protection for takeout employees, this paper compared the occupational injuries of takeout employees before and after the optimization of employment protection, as well as the impact on takeout employees. In order to effectively compare the optimization effect of employment protection for employees engaged in outbound sales, the main outbound sales platforms in China were analyzed. Meituan takeout and ELEME takeout occupy the takeout market in China. Therefore, this paper randomly selected 100 Meituan takeout employees and 100 Lulemao takeout employees as the objects of experimental comparison before and after the optimization of employment protection.

The takeout employees selected in the experiment did not know about this experiment in advance and worked normally during the experiment. The comparison points before and after the optimization of the employment protection for takeout employees are: the rationality of the delivery management system, the comfort of the working environment, the work intensity and the psychological pressure. In order to make the comparison before and after the optimization of the employment protection of takeout employees more sufficient, the experiment conducted data statistics on selected experimental personnel 5 months before and 5 months after the optimization of the employment protection. The frequency of data statistics was once a month. The purpose of this was to make the data of the optimization of the employment protection of takeout employees more detailed, so as to prevent external factors from interfering with the experiment.

## 4. Results of optimization of employment protection for takeout employees

### 4.1. Rationality of delivery management system

**Figure 5:**
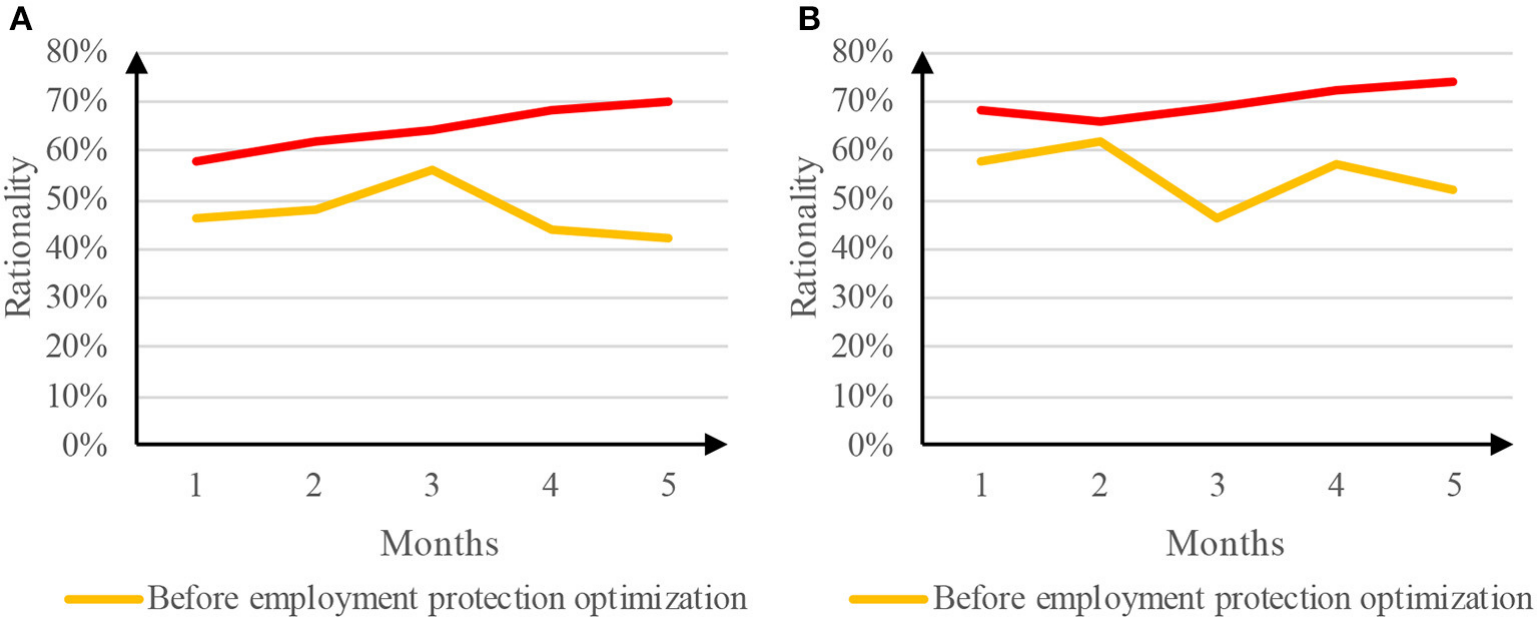
Comparison of rationality of delivery management system. **(A)** Rationality of Meituan takeout distribution management system. **(B)** Rationality of ELEME takeout distribution management system.

The traditional delivery management system is mainly based on the quantity of delivery orders, which allows the delivery practitioners to deliver as many goods as possible in a short time. The rationality of the delivery management system is closely related to the occupational injuries of the delivery employees. The rationality of the delivery management system before and after the optimization of employment protection was compared. The comparison results are shown in Figure 5.

Figure 5A is a comparison of the rationality of the Meituan delivery management system before and after the optimization of employment protection. The rationality of the delivery management system before the optimization of employment protection reached a minimum of 42% in the fifth month and a maximum of 56% in the third month. The rationality of the average delivery management system was 47.2%. However, the rationality of the delivery management system after the optimization of employment protection was continuously improved, from 58% in the first month to 70% in the fifth month, and the average rationality of the delivery management system was 64.4%. Figure 5B is a comparison of the rationality of the delivery management system before and after the optimization of employment protection. The rationality of the delivery management system before the optimization of employment protection fluctuated continuously during the experimental period; it reached a minimum of 46% in the third month and a maximum of 62% in the second month. The rationality of the average delivery management system was 55.0%. The rationality of the delivery management system after the optimization of employment protection reached a minimum of 66% in the second month and a maximum of 74% in the fifth month. The rationality of the average delivery management system was 69.8%. The continuous improvement of the rationality of the delivery management system after the optimization of employment protection may be due to the reduction of the delivery volume requirements of the delivery platform. Therefore, the optimization of the employment protection of takeout employees can effectively improve the rationality of the delivery management system.

### 4.2. Comfort of working environment

**Figure 6:**
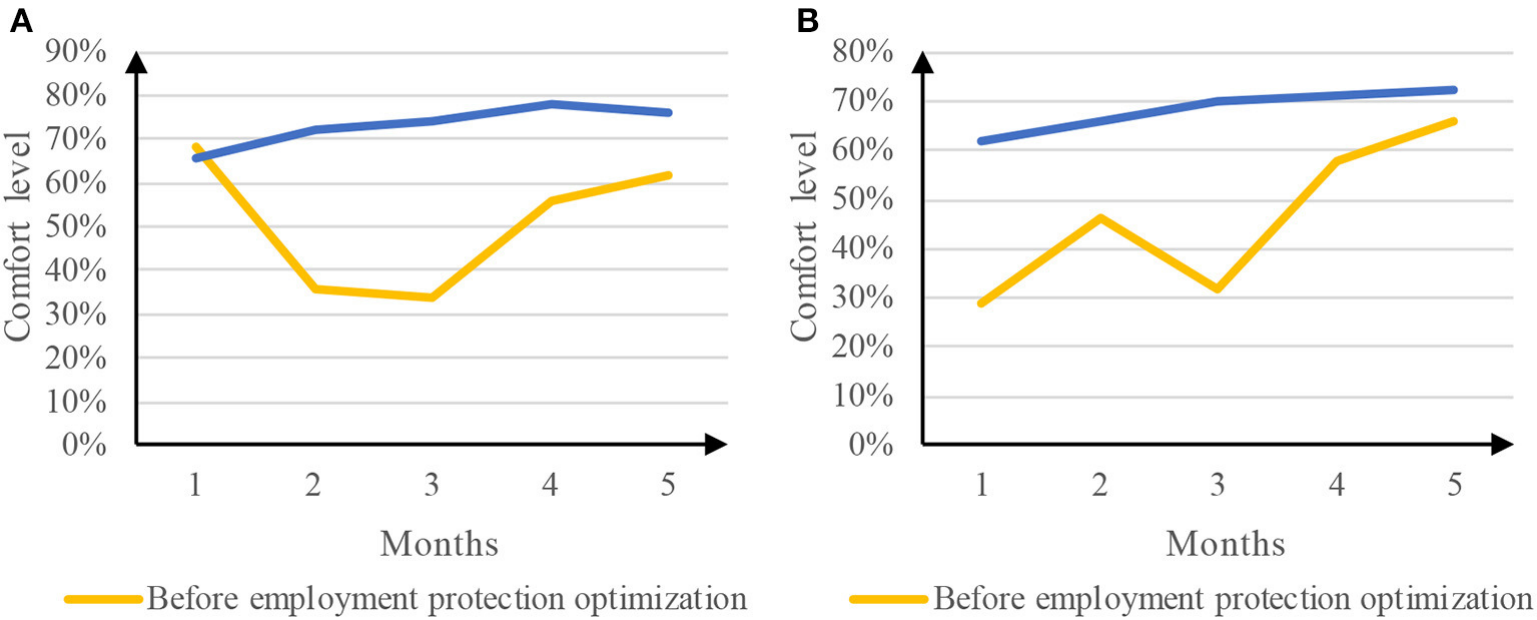
Comparison results of working environment comfort. **(A)** Comfort of Meituan takeout working environment. **(B)** Comfort of ELEME takeout working environment.

The working environment of takeout employees is the main cause of occupational injury, and takeout distribution under high temperature and strong wind is prone to risk. The comfort of working environment before and after the optimization of employment protection was compared. The comparison results are shown in Figure 6.

Figure 6A is a comparison of the comfort level of Meituan takeout working environment before and after the optimization of employment protection. Among them, the comfort level of the takeout working environment before the optimization of employment protection reached a minimum of 34% in the third month and a maximum of 68% in the first month. The average comfort level of the takeout working environment was 51.2%. The poor comfort of the takeout working environment in the second and third months may be caused by bad weather. After the optimization of employment protection, the comfort level of the takeout working environment reached a minimum of 66% in the first month and a maximum of 78% in the fourth month. The average comfort level of the takeout working environment was 73.2%. Figure 6B is a comparison of the comfort level of the takeout working environment before and after the optimization of employment protection. Before the optimization of employment protection, the comfort level of takeout working environment reached a minimum of 29% in the first month and a maximum of 66% in the fifth month. The average comfort level of takeout working environment was 46.2%. After the optimization of employment protection, the comfort level of the takeout working environment was constantly improving, The comfort level of the takeout working environment increased from 62% in the first month to 72% in the fifth month, and the average comfort level of the takeout working environment was 68.2%. Therefore, employment protection optimization can effectively improve the comfort of the takeout working environment.

### 4.3. Working intensity

**Figure 7:**
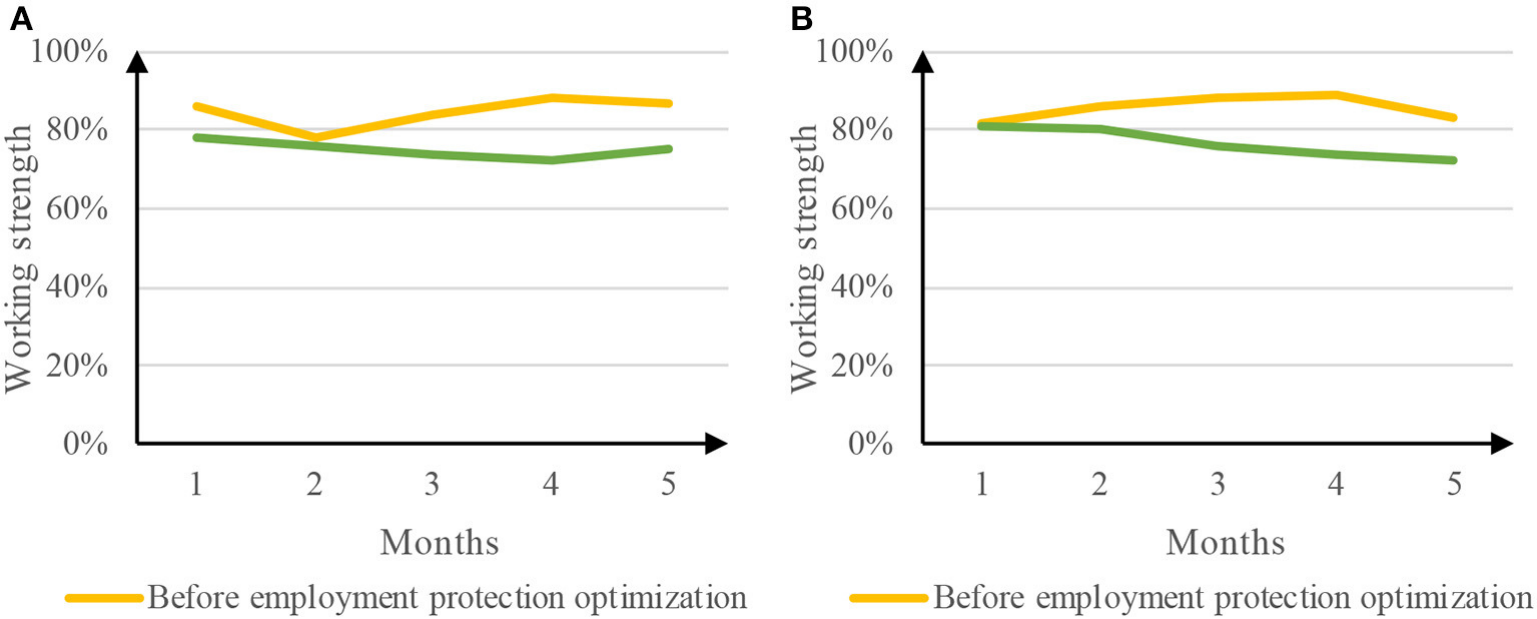
Comparison results of work intensity. **(A)** Work intensity of Meituan takeout. **(B)** Work intensity of ELEME takeout.

The work intensity of takeout employees is very high, and the daily delivery volume is very large. In order to complete the task, the delivery employees must improve the delivery speed, which causes many takeout employees to be in danger in the delivery process. The work intensity before and after the optimization of employment protection was compared. The comparison results are shown in Figure 7.

Figure 7A is a comparison of the intensity of Meituan takeout before and after the optimization of employment protection. The takeout work intensity before the optimization of employment protection reached a minimum of 78% in the second month and a maximum of 88% in the fourth month, with an average work intensity of 84.6%. After the optimization of employment protection, the takeout work intensity reached a minimum of 72% in the fourth month and a maximum of 78% in the first month, with an average work intensity of 75.0%. Figure 7B is a comparison of the intensity of takeout before and after the optimization of employment protection. Before the optimization of employment protection, the takeout work intensity reached a minimum of 82% in the first month and a maximum of 89% in the fourth month, with an average work intensity of 85.6%. After the optimization of employment protection, the intensity of takeout was decreasing, from 81% in the first month to 72% in the fifth month, with an average intensity of 76.6%. Therefore, employment protection optimization, such as strengthening employment relationship, can effectively reduce the work intensity of takeout employees.

### 4.4. Psychological pressure

**Figure 8:**
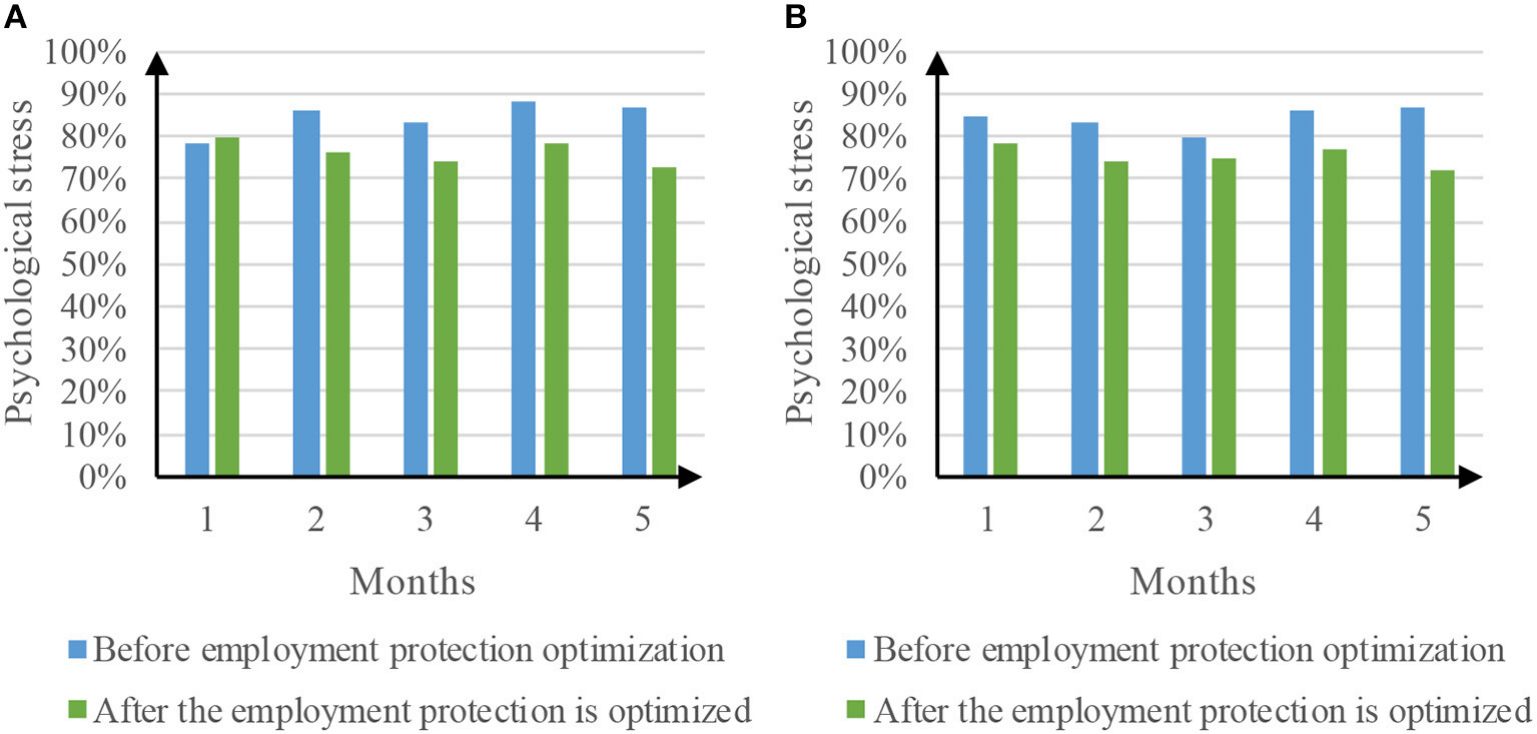
Comparison results of psychological stress. **(A)** Psychological pressure of Meituan takeout. **(B)** Psychological pressure of ELEME takeout.

The performance of takeout employees is directly linked to the evaluation of consumers, which also makes the psychological pressure of takeout employees very high. Efforts are made to improve the delivery speed to obtain the praise of consumers, and the negative evaluation of consumers has a great impact on takeout employees. Long term psychological stress would make the takeout workers become depressed, and they often do not eat and drink on time in order to rush, which is very easy to cause stomach disease. The comparison of psychological pressure before and after the optimization of employment protection is shown in Figure 8.

Figure 8A is a comparison of the psychological pressure of Meituan takeout before and after the optimization of employment protection. Before the optimization of employment protection, the psychological pressure of takeout work reached a minimum of 78% in the first month and a maximum of 88% in the fourth month. The average psychological pressure of work was 84.4%. After the optimization of employment protection, the psychological pressure of takeout work reached a minimum of 73% in the fifth month and a maximum of 80% in the first month. The average psychological pressure of work was 76.2%. Figure 8B shows the comparison of psychological pressure of taking out food before and after the optimization of employment protection. Before the optimization of employment protection, the psychological pressure of takeout work reached a minimum of 80% in the third month and a maximum of 87% in the fifth month. The average psychological pressure of work was 84.2%. After the optimization of employment protection, the psychological pressure of takeout work reached a minimum of 72% in the fifth month and a maximum of 78% in the first month. The average psychological pressure of work was 75.2%. Therefore, the optimization of employment protection through service evaluation mechanism can significantly reduce the psychological pressure of takeout employees.

## 5. Conclusions

In recent years, the accidents of takeout workers have occurred frequently, and the occupational injuries and employment security of takeout workers have received extensive attention. As a service industry, the core of takeout industry is to focus on the rights and interests of consumers, which makes takeout practitioners very hasty when distributing takeout food. Accidents are very easy to occur in the road sections with complex traffic, and the long-term and compact distribution tasks cause great physical and psychological damage to the takeout employees. This paper analyzed the professional characteristics of the takeout workers, and optimized the social security, employment relationship and service evaluation mechanism of the takeout workers. This paper also used the AHP to build an evaluation system for the optimization of the employment protection of takeout workers, and compared it before and after the optimization. The results showed that the optimization of employment protection can effectively improve the rationality of the delivery management system, the comfort of the working environment, and reduce the work intensity and psychological pressure at work. The optimization of employment protection can reduce the occupational injury of takeout workers and ensure the occupational safety of takeout workers. However, this article analyzes the changes before and after the employment protection of the takeaway workers from four aspects, and makes a reasonable comparison of the work intensity of the takeaway workers, but lacks a comparison of the occupational injury of the takeaway workers. Therefore, through the questionnaire survey of more takeout employees, the analysis of objective indicators that can evaluate the optimization effect of employment protection for take-away workers would be the direction of future research.

## Data availability statement

The original contributions presented in the study are included in the article/supplementary material, further inquiries can be directed to the corresponding author.

## Author contributions

All authors listed have made a substantial, direct, and intellectual contribution to the work and approved it for publication.
